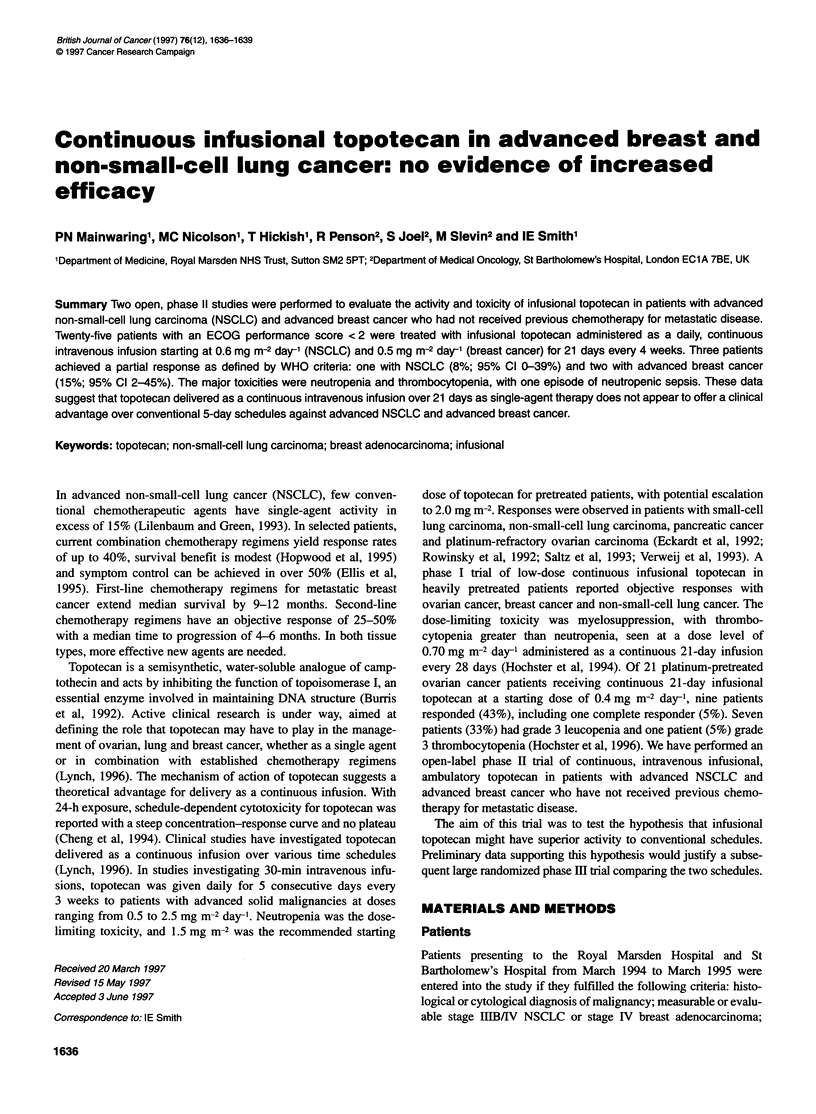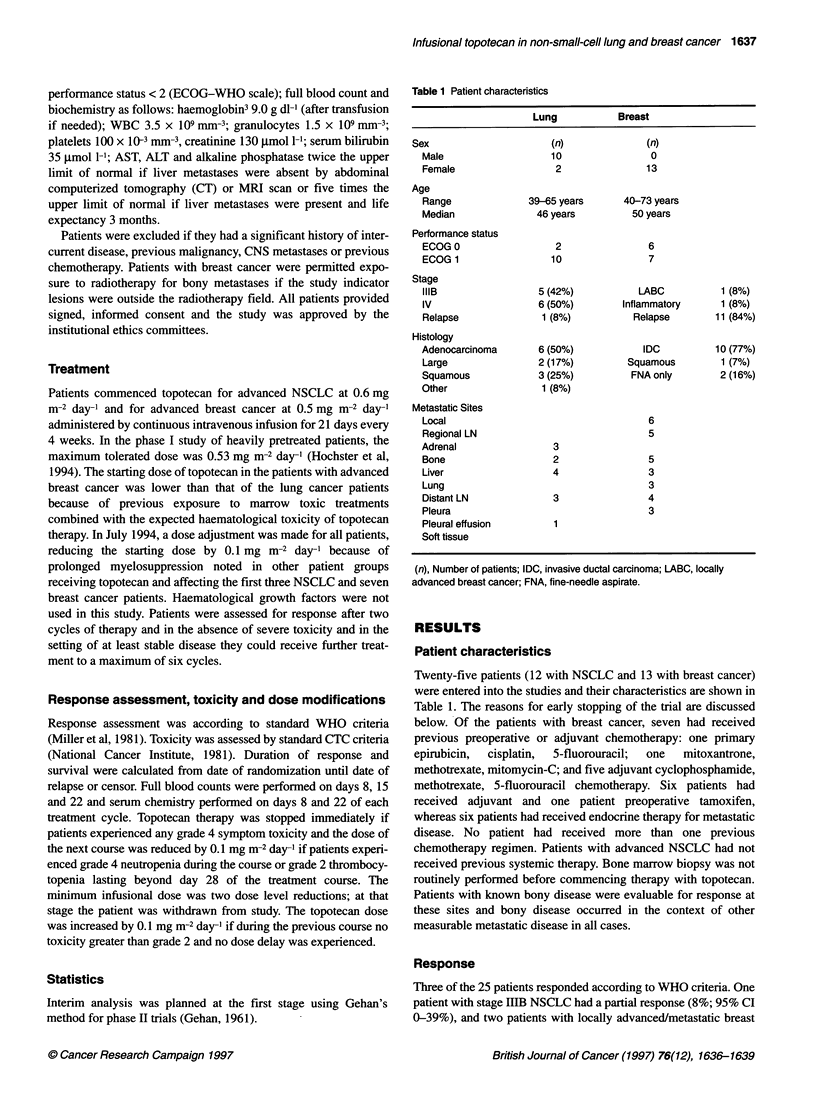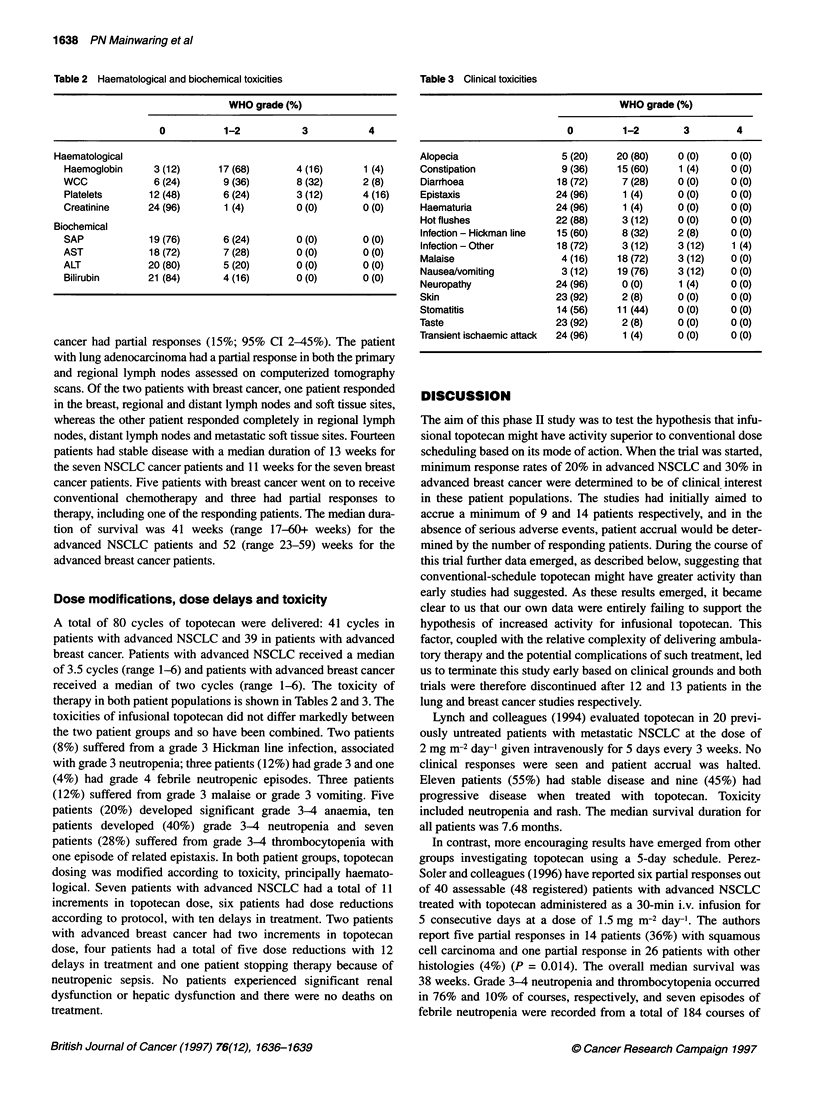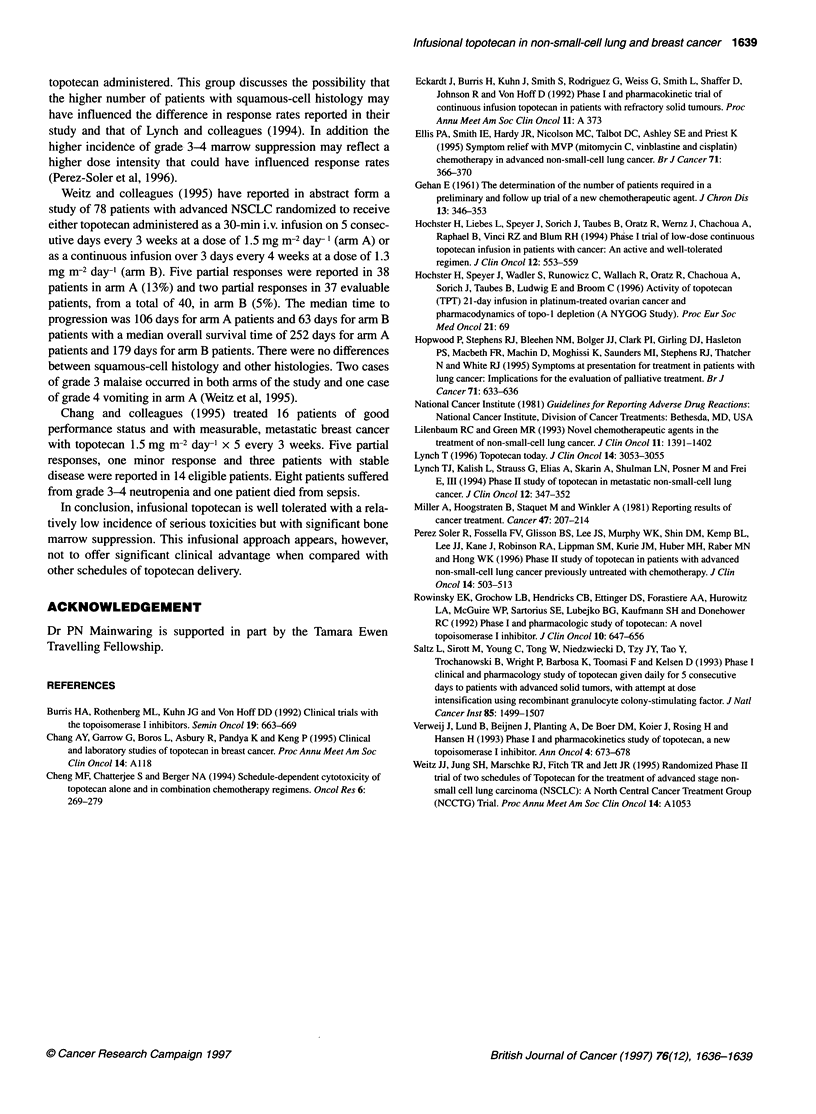# Continuous infusional topotecan in advanced breast and non-small-cell lung cancer: no evidence of increased efficacy.

**DOI:** 10.1038/bjc.1997.609

**Published:** 1997

**Authors:** P. N. Mainwaring, M. C. Nicolson, T. Hickish, R. Penson, S. Joel, M. Slevin, I. E. Smith

**Affiliations:** Department of Medicine, Royal Marsden NHS Trust, Sutton, UK.

## Abstract

Two open, phase II studies were performed to evaluate the activity and toxicity of infusional topotecan in patients with advanced non-small-cell lung carcinoma (NSCLC) and advanced breast cancer who had not received previous chemotherapy for metastatic disease. Twenty-five patients with an ECOG performance score < 2 were treated with infusional topotecan administered as a daily, continuous intravenous infusion starting at 0.6 mg m(-2) day(-1) (NSCLC) and 0.5 mg m(-2) day(-1) (breast cancer) for 21 days every 4 weeks. Three patients achieved a partial response as defined by WHO criteria: one with NSCLC (8%; 95% CI 0-39%) and two with advanced breast cancer (15%; 95% CI 2-45%). The major toxicities were neutropenia and thrombocytopenia, with one episode of neutropenic sepsis. These data suggest that topotecan delivered as a continuous intravenous infusion over 21 days as single-agent therapy does not appear to offer a clinical advantage over conventional 5-day schedules against advanced NSCLC and advanced breast cancer.


British Journal of Cancer (1997) 76(12), 1636–1639
© 1997 Cancer Research Campaign

### Continuous infusional topotecan in advanced breast and non-small-cell lung cancer: no evidence of increased efficacy

**PN Mainwaring[1], MC Nicolson[1], T Hickish[1], R Penson[2], S Joel[2], M Slevin[2] and IE Smith[1]**

[1]Department of Medicine, Royal Marsden NHS Trust, Sutton SM2 5PT; [2]Department of Medical Oncology, St Bartholomew's Hospital, London EC1A 7BE, UK

**Summary** Two open, phase II studies were performed to evaluate the activity and toxicity of infusional topotecan in patients with advanced non-small-cell lung carcinoma (NSCLC) and advanced breast cancer who had not received previous chemotherapy for metastatic disease. Twenty-five patients with an ECOG performance score < 2 were treated with infusional topotecan administered as a daily, continuous intravenous infusion starting at 0.6 mg $m^{-2}$ $day^{-1}$ (NSCLC) and 0.5 mg $m^{-2}$ $day^{-1}$ (breast cancer) for 21 days every 4 weeks. Three patients achieved a partial response as defined by WHO criteria: one with NSCLC (8%; 95% CI 0–39%) and two with advanced breast cancer (15%; 95% CI 2–45%). The major toxicities were neutropenia and thrombocytopenia, with one episode of neutropenic sepsis. These data suggest that topotecan delivered as a continuous intravenous infusion over 21 days as single-agent therapy does not appear to offer a clinical advantage over conventional 5-day schedules against advanced NSCLC and advanced breast cancer.

**Keywords:** topotecan; non-small-cell lung carcinoma; breast adenocarcinoma; infusional

In advanced non-small-cell lung cancer (NSCLC), few conventional chemotherapeutic agents have single-agent activity in excess of 15% (Lilenbaum and Green, 1993). In selected patients, current combination chemotherapy regimens yield response rates of up to 40%, survival benefit is modest (Hopwood et al, 1995) and symptom control can be achieved in over 50% (Ellis et al, 1995). First-line chemotherapy regimens for metastatic breast cancer extend median survival by 9–12 months. Second-line chemotherapy regimens have an objective response of 25–50% with a median time to progression of 4–6 months. In both tissue types, more effective new agents are needed.

Topotecan is a semisynthetic, water-soluble analogue of camptothecin and acts by inhibiting the function of topoisomerase I, an essential enzyme involved in maintaining DNA structure (Burris et al, 1992). Active clinical research is under way, aimed at defining the role that topotecan may have to play in the management of ovarian, lung and breast cancer, whether as a single agent or in combination with established chemotherapy regimens (Lynch, 1996). The mechanism of action of topotecan suggests a theoretical advantage for delivery as a continuous infusion. With 24-h exposure, schedule-dependent cytotoxicity for topotecan was reported with a steep concentration–response curve and no plateau (Cheng et al, 1994). Clinical studies have investigated topotecan delivered as a continuous infusion over various time schedules (Lynch, 1996). In studies investigating 30-min intravenous infusions, topotecan was given daily for 5 consecutive days every 3 weeks to patients with advanced solid malignancies at doses ranging from 0.5 to 2.5 mg $m^{-2}$ $day^{-1}$. Neutropenia was the dose-limiting toxicity, and 1.5 mg $m^{-2}$ was the recommended starting dose of topotecan for pretreated patients, with potential escalation to 2.0 mg $m^{-2}$. Responses were observed in patients with small-cell lung carcinoma, non-small-cell lung carcinoma, pancreatic cancer and platinum-refractory ovarian carcinoma (Eckardt et al, 1992; Rowinsky et al, 1992; Saltz et al, 1993; Verweij et al, 1993). A phase I trial of low-dose continuous infusional topotecan in heavily pretreated patients reported objective responses with ovarian cancer, breast cancer and non-small-cell lung cancer. The dose-limiting toxicity was myelosuppression, with thrombocytopenia greater than neutropenia, seen at a dose level of 0.70 mg $m^{-2}$ $day^{-1}$ administered as a continuous 21-day infusion every 28 days (Hochster et al, 1994). Of 21 platinum-pretreated ovarian cancer patients receiving continuous 21-day infusional topotecan at a starting dose of 0.4 mg $m^{-2}$ $day^{-1}$, nine patients responded (43%), including one complete responder (5%). Seven patients (33%) had grade 3 leucopenia and one patient (5%) grade 3 thrombocytopenia (Hochster et al, 1996). We have performed an open-label phase II trial of continuous, intravenous infusional, ambulatory topotecan in patients with advanced NSCLC and advanced breast cancer who have not received previous chemotherapy for metastatic disease.

The aim of this trial was to test the hypothesis that infusional topotecan might have superior activity to conventional schedules. Preliminary data supporting this hypothesis would justify a subsequent large randomized phase III trial comparing the two schedules.

### MATERIALS AND METHODS

### Patients

Patients presenting to the Royal Marsden Hospital and St Bartholomew's Hospital from March 1994 to March 1995 were entered into the study if they fulfilled the following criteria: histological or cytological diagnosis of malignancy; measurable or evaluable stage IIIB/IV NSCLC or stage IV breast adenocarcinoma;

Received 20 March 1997
Revised 15 May 1997
Accepted 3 June 1997

Correspondence to: IE Smith performance status < 2 (ECOG–WHO scale); full blood count and biochemistry as follows: haemoglobin$^{3}$ 9.0 g dl$^{-1}$ (after transfusion if needed); WBC 3.5 × $10^9$ mm$^{-3}$; granulocytes 1.5 × $10^9$ mm$^{-3}$; platelets 100 × $10^{-3}$ mm$^{-3}$, creatinine 130 μmol l$^{-1}$; serum bilirubin 35 μmol l$^{-1}$; AST, ALT and alkaline phosphatase twice the upper limit of normal if liver metastases were absent by abdominal computerized tomography (CT) or MRI scan or five times the upper limit of normal if liver metastases were present and life expectancy 3 months.

Patients were excluded if they had a significant history of intercurrent disease, previous malignancy, CNS metastases or previous chemotherapy. Patients with breast cancer were permitted exposure to radiotherapy for bony metastases if the study indicator lesions were outside the radiotherapy field. All patients provided signed, informed consent and the study was approved by the institutional ethics committees.

### Treatment

Patients commenced topotecan for advanced NSCLC at 0.6 mg m$^{-2}$ day$^{-1}$ and for advanced breast cancer at 0.5 mg m$^{-2}$ day$^{-1}$ administered by continuous intravenous infusion for 21 days every 4 weeks. In the phase I study of heavily pretreated patients, the maximum tolerated dose was 0.53 mg m$^{-2}$ day$^{-1}$ (Hochster et al, 1994). The starting dose of topotecan in the patients with advanced breast cancer was lower than that of the lung cancer patients because of previous exposure to marrow toxic treatments combined with the expected haematological toxicity of topotecan therapy. In July 1994, a dose adjustment was made for all patients, reducing the starting dose by 0.1 mg m$^{-2}$ day$^{-1}$ because of prolonged myelosuppression noted in other patient groups receiving topotecan and affecting the first three NSCLC and seven breast cancer patients. Haematological growth factors were not used in this study. Patients were assessed for response after two cycles of therapy and in the absence of severe toxicity and in the setting of at least stable disease they could receive further treatment to a maximum of six cycles.

### Response assessment, toxicity and dose modifications

Response assessment was according to standard WHO criteria (Miller et al, 1981). Toxicity was assessed by standard CTC criteria (National Cancer Institute, 1981). Duration of response and survival were calculated from date of randomization until date of relapse or censor. Full blood counts were performed on days 8, 15 and 22 and serum chemistry performed on days 8 and 22 of each treatment cycle. Topotecan therapy was stopped immediately if patients experienced any grade 4 symptom toxicity and the dose of the next course was reduced by 0.1 mg m$^{-2}$ day$^{-1}$ if patients experienced grade 4 neutropenia during the course or grade 2 thrombocytopenia lasting beyond day 28 of the treatment course. The minimum infusional dose was two dose level reductions; at that stage the patient was withdrawn from study. The topotecan dose was increased by 0.1 mg m$^{-2}$ day$^{-1}$ if during the previous course no toxicity greater than grade 2 and no dose delay was experienced.

### Statistics

Interim analysis was planned at the first stage using Gehan's method for phase II trials (Gehan, 1961).

**Table 1** Patient characteristics

| | Lung | Breast | |
|---|---|---|---|
| Sex | (*n*) | (*n*) | |
| Male | 10 | 0 | |
| Female | 2 | 13 | |
| Age | | | |
| Range | 39–65 years | 40–73 years | |
| Median | 46 years | 50 years | |
| Performance status | | | |
| ECOG 0 | 2 | 6 | |
| ECOG 1 | 10 | 7 | |
| Stage | | | |
| IIIB | 5 (42%) | LABC | 1 (8%) |
| IV | 6 (50%) | Inflammatory | 1 (8%) |
| Relapse | 1 (8%) | Relapse | 11 (84%) |
| Histology | | | |
| Adenocarcinoma | 6 (50%) | IDC | 10 (77%) |
| Large | 2 (17%) | Squamous | 1 (7%) |
| Squamous | 3 (25%) | FNA only | 2 (16%) |
| Other | 1 (8%) | | |
| Metastatic Sites | | | |
| Local | | 6 | |
| Regional LN | | 5 | |
| Adrenal | 3 | | |
| Bone | 2 | 5 | |
| Liver | 4 | 3 | |
| Lung | | 3 | |
| Distant LN | 3 | 4 | |
| Pleura | | 3 | |
| Pleural effusion | 1 | | |
| Soft tissue | | | |

(*n*), Number of patients; IDC, invasive ductal carcinoma; LABC, locally advanced breast cancer; FNA, fine-needle aspirate.

### RESULTS

### Patient characteristics

Twenty-five patients (12 with NSCLC and 13 with breast cancer) were entered into the studies and their characteristics are shown in Table 1. The reasons for early stopping of the trial are discussed below. Of the patients with breast cancer, seven had received previous preoperative or adjuvant chemotherapy: one primary epirubicin, cisplatin, 5-fluorouracil; one mitoxantrone, methotrexate, mitomycin-C; and five adjuvant cyclophosphamide, methotrexate, 5-fluorouracil chemotherapy. Six patients had received adjuvant and one patient preoperative tamoxifen, whereas six patients had received endocrine therapy for metastatic disease. No patient had received more than one previous chemotherapy regimen. Patients with advanced NSCLC had not received previous systemic therapy. Bone marrow biopsy was not routinely performed before commencing therapy with topotecan. Patients with known bony disease were evaluable for response at these sites and bony disease occurred in the context of other measurable metastatic disease in all cases.

### Response

Three of the 25 patients responded according to WHO criteria. One patient with stage IIIB NSCLC had a partial response (8%; 95% CI 0–39%), and two patients with locally advanced/metastatic breast

*© Cancer Research Campaign 1997*

Table 2 Haematological and biochemical toxicities

| | WHO grade (%) | | | |
|---|---|---|---|---|
| | 0 | 1–2 | 3 | 4 |
| Haematological | | | | |
| Haemoglobin | 3 (12) | 17 (68) | 4 (16) | 1 (4) |
| WCC | 6 (24) | 9 (36) | 8 (32) | 2 (8) |
| Platelets | 12 (48) | 6 (24) | 3 (12) | 4 (16) |
| Creatinine | 24 (96) | 1 (4) | 0 (0) | 0 (0) |
| Biochemical | | | | |
| SAP | 19 (76) | 6 (24) | 0 (0) | 0 (0) |
| AST | 18 (72) | 7 (28) | 0 (0) | 0 (0) |
| ALT | 20 (80) | 5 (20) | 0 (0) | 0 (0) |
| Bilirubin | 21 (84) | 4 (16) | 0 (0) | 0 (0) |

cancer had partial responses (15%; 95% CI 2–45%). The patient with lung adenocarcinoma had a partial response in both the primary and regional lymph nodes assessed on computerized tomography scans. Of the two patients with breast cancer, one patient responded in the breast, regional and distant lymph nodes and soft tissue sites, whereas the other patient responded completely in regional lymph nodes, distant lymph nodes and metastatic soft tissue sites. Fourteen patients had stable disease with a median duration of 13 weeks for the seven NSCLC cancer patients and 11 weeks for the seven breast cancer patients. Five patients with breast cancer went on to receive conventional chemotherapy and three had partial responses to therapy, including one of the responding patients. The median duration of survival was 41 weeks (range 17–60+ weeks) for the advanced NSCLC patients and 52 (range 23–59) weeks for the advanced breast cancer patients.

### Dose modifications, dose delays and toxicity

A total of 80 cycles of topotecan were delivered: 41 cycles in patients with advanced NSCLC and 39 in patients with advanced breast cancer. Patients with advanced NSCLC received a median of 3.5 cycles (range 1–6) and patients with advanced breast cancer received a median of two cycles (range 1–6). The toxicity of therapy in both patient populations is shown in Tables 2 and 3. The toxicities of infusional topotecan did not differ markedly between the two patient groups and so have been combined. Two patients (8%) suffered from a grade 3 Hickman line infection, associated with grade 3 neutropenia; three patients (12%) had grade 3 and one (4%) had grade 4 febrile neutropenic episodes. Three patients (12%) suffered from grade 3 malaise or grade 3 vomiting. Five patients (20%) developed significant grade 3–4 anaemia, ten patients developed (40%) grade 3–4 neutropenia and seven patients (28%) suffered from grade 3–4 thrombocytopenia with one episode of related epistaxis. In both patient groups, topotecan dosing was modified according to toxicity, principally haematological. Seven patients with advanced NSCLC had a total of 11 increments in topotecan dose, six patients had dose reductions according to protocol, with ten delays in treatment. Two patients with advanced breast cancer had two increments in topotecan dose, four patients had a total of five dose reductions with 12 delays in treatment and one patient stopping therapy because of neutropenic sepsis. No patients experienced significant renal dysfunction or hepatic dysfunction and there were no deaths on treatment.

Table 3 Clinical toxicities

| | WHO grade (%) | | | |
|---|---|---|---|---|
| | 0 | 1–2 | 3 | 4 |
| Alopecia | 5 (20) | 20 (80) | 0 (0) | 0 (0) |
| Constipation | 9 (36) | 15 (60) | 1 (4) | 0 (0) |
| Diarrhoea | 18 (72) | 7 (28) | 0 (0) | 0 (0) |
| Epistaxis | 24 (96) | 1 (4) | 0 (0) | 0 (0) |
| Haematuria | 24 (96) | 1 (4) | 0 (0) | 0 (0) |
| Hot flushes | 22 (88) | 3 (12) | 0 (0) | 0 (0) |
| Infection – Hickman line | 15 (60) | 8 (32) | 2 (8) | 0 (0) |
| Infection – Other | 18 (72) | 3 (12) | 3 (12) | 1 (4) |
| Malaise | 4 (16) | 18 (72) | 3 (12) | 0 (0) |
| Nausea/vomiting | 3 (12) | 19 (76) | 3 (12) | 0 (0) |
| Neuropathy | 24 (96) | 0 (0) | 1 (4) | 0 (0) |
| Skin | 23 (92) | 2 (8) | 0 (0) | 0 (0) |
| Stomatitis | 14 (56) | 11 (44) | 0 (0) | 0 (0) |
| Taste | 23 (92) | 2 (8) | 0 (0) | 0 (0) |
| Transient ischaemic attack | 24 (96) | 1 (4) | 0 (0) | 0 (0) |

### DISCUSSION

The aim of this phase II study was to test the hypothesis that infusional topotecan might have activity superior to conventional dose scheduling based on its mode of action. When the trial was started, minimum response rates of 20% in advanced NSCLC and 30% in advanced breast cancer were determined to be of clinical interest in these patient populations. The studies had initially aimed to accrue a minimum of 9 and 14 patients respectively, and in the absence of serious adverse events, patient accrual would be determined by the number of responding patients. During the course of this trial further data emerged, as described below, suggesting that conventional-schedule topotecan might have greater activity than early studies had suggested. As these results emerged, it became clear to us that our own data were entirely failing to support the hypothesis of increased activity for infusional topotecan. This factor, coupled with the relative complexity of delivering ambulatory therapy and the potential complications of such treatment, led us to terminate this study early based on clinical grounds and both trials were therefore discontinued after 12 and 13 patients in the lung and breast cancer studies respectively.

Lynch and colleagues (1994) evaluated topotecan in 20 previously untreated patients with metastatic NSCLC at the dose of 2 mg $m^{-2}$ $day^{-1}$ given intravenously for 5 days every 3 weeks. No clinical responses were seen and patient accrual was halted. Eleven patients (55%) had stable disease and nine (45%) had progressive disease when treated with topotecan. Toxicity included neutropenia and rash. The median survival duration for all patients was 7.6 months.

In contrast, more encouraging results have emerged from other groups investigating topotecan using a 5-day schedule. Perez-Soler and colleagues (1996) have reported six partial responses out of 40 assessable (48 registered) patients with advanced NSCLC treated with topotecan administered as a 30-min i.v. infusion for 5 consecutive days at a dose of 1.5 mg $m^{-2}$ $day^{-1}$. The authors report five partial responses in 14 patients (36%) with squamous cell carcinoma and one partial response in 26 patients with other histologies (4%) ($P$ = 0.014). The overall median survival was 38 weeks. Grade 3–4 neutropenia and thrombocytopenia occurred in 76% and 10% of courses, respectively, and seven episodes of febrile neutropenia were recorded from a total of 184 courses of

© Cancer Research Campaign 1997

topotecan administered. This group discusses the possibility that the higher number of patients with squamous-cell histology may have influenced the difference in response rates reported in their study and that of Lynch and colleagues (1994). In addition the higher incidence of grade 3–4 marrow suppression may reflect a higher dose intensity that could have influenced response rates (Perez-Soler et al, 1996).

Weitz and colleagues (1995) have reported in abstract form a study of 78 patients with advanced NSCLC randomized to receive either topotecan administered as a 30-min i.v. infusion on 5 consecutive days every 3 weeks at a dose of 1.5 mg $m^{-2}$ $day^{-1}$ (arm A) or as a continuous infusion over 3 days every 4 weeks at a dose of 1.3 mg $m^{-2}$ $day^{-1}$ (arm B). Five partial responses were reported in 38 patients in arm A (13%) and two partial responses in 37 evaluable patients, from a total of 40, in arm B (5%). The median time to progression was 106 days for arm A patients and 63 days for arm B patients with a median overall survival time of 252 days for arm A patients and 179 days for arm B patients. There were no differences between squamous-cell histology and other histologies. Two cases of grade 3 malaise occurred in both arms of the study and one case of grade 4 vomiting in arm A (Weitz et al, 1995).

Chang and colleagues (1995) treated 16 patients of good performance status and with measurable, metastatic breast cancer with topotecan 1.5 mg $m^{-2}$ $day^{-1}$ × 5 every 3 weeks. Five partial responses, one minor response and three patients with stable disease were reported in 14 eligible patients. Eight patients suffered from grade 3–4 neutropenia and one patient died from sepsis.

In conclusion, infusional topotecan is well tolerated with a relatively low incidence of serious toxicities but with significant bone marrow suppression. This infusional approach appears, however, not to offer significant clinical advantage when compared with other schedules of topotecan delivery.

### ACKNOWLEDGEMENT

Dr PN Mainwaring is supported in part by the Tamara Ewen Travelling Fellowship.

### REFERENCES

Burris HA, Rothenberg ML, Kuhn JG and Von Hoff DD (1992) Clinical trials with the topoisomerase I inhibitors. *Semin Oncol* **19**: 663–669

Chang AY, Garrow G, Boros L, Asbury R, Pandya K and Keng P (1995) Clinical and laboratory studies of topotecan in breast cancer. *Proc Annu Meet Am Soc Clin Oncol* **14**: A118

Cheng MF, Chatterjee S and Berger NA (1994) Schedule-dependent cytotoxicity of topotecan alone and in combination chemotherapy regimens. *Oncol Res* **6**: 269–279

Eckardt J, Burris H, Kuhn J, Smith S, Rodriguez G, Weiss G, Smith L, Shaffer D, Johnson R and Von Hoff D (1992) Phase I and pharmacokinetic trial of continuous infusion topotecan in patients with refractory solid tumours. *Proc Annu Meet Am Soc Clin Oncol* **11**: A 373

Ellis PA, Smith IE, Hardy JR, Nicolson MC, Talbot DC, Ashley SE and Priest K (1995) Symptom relief with MVP (mitomycin C, vinblastine and cisplatin) chemotherapy in advanced non-small-cell lung cancer. *Br J Cancer* **71**: 366–370

Gehan E (1961) The determination of the number of patients required in a preliminary and follow up trial of a new chemotherapeutic agent. *J Chron Dis* **13**: 346–353

Hochster H, Liebes L, Speyer J, Sorich J, Taubes B, Oratz R, Wernz J, Chachoua A, Raphael B, Vinci RZ and Blum RH (1994) Phase I trial of low-dose continuous topotecan infusion in patients with cancer: An active and well-tolerated regimen. *J Clin Oncol* **12**: 553–559

Hochster H, Speyer J, Wadler S, Runowicz C, Wallach R, Oratz R, Chachoua A, Sorich J, Taubes B, Ludwig E and Broom C (1996) Activity of topotecan (TPT) 21-day infusion in platinum-treated ovarian cancer and pharmacodynamics of topo-1 depletion (A NYGOG Study). *Proc Eur Soc Med Oncol* **21**: 69

Hopwood P, Stephens RJ, Bleehen NM, Bolger JJ, Clark PI, Girling DJ, Hasleton PS, Macbeth FR, Machin D, Moghissi K, Saunders MI, Stephens RJ, Thatcher N and White RJ (1995) Symptoms at presentation for treatment in patients with lung cancer: Implications for the evaluation of palliative treatment. *Br J Cancer* **71**: 633–636

National Cancer Institute (1981) *Guidelines for Reporting Adverse Drug Reactions*: National Cancer Institute, Division of Cancer Treatments: Bethesda, MD, USA

Lilenbaum RC and Green MR (1993) Novel chemotherapeutic agents in the treatment of non-small-cell lung cancer. *J Clin Oncol* **11**: 1391–1402

Lynch T (1996) Topotecan today. *J Clin Oncol* **14**: 3053–3055

Lynch TJ, Kalish L, Strauss G, Elias A, Skarin A, Shulman LN, Posner M and Frei E, III (1994) Phase II study of topotecan in metastatic non-small-cell lung cancer. *J Clin Oncol* **12**: 347–352

Miller A, Hoogstraten B, Staquet M and Winkler A (1981) Reporting results of cancer treatment. *Cancer* **47**: 207–214

Perez Soler R, Fossella FV, Glisson BS, Lee JS, Murphy WK, Shin DM, Kemp BL, Lee JJ, Kane J, Robinson RA, Lippman SM, Kurie JM, Huber MH, Raber MN and Hong WK (1996) Phase II study of topotecan in patients with advanced non-small-cell lung cancer previously untreated with chemotherapy. *J Clin Oncol* **14**: 503–513

Rowinsky EK, Grochow LB, Hendricks CB, Ettinger DS, Forastiere AA, Hurowitz LA, McGuire WP, Sartorius SE, Lubejko BG, Kaufmann SH and Donehower RC (1992) Phase I and pharmacologic study of topotecan: A novel topoisomerase I inhibitor. *J Clin Oncol* **10**: 647–656

Saltz L, Sirott M, Young C, Tong W, Niedzwiecki D, Tzy JY, Tao Y, Trochanowski B, Wright P, Barbosa K, Toomasi F and Kelsen D (1993) Phase I clinical and pharmacology study of topotecan given daily for 5 consecutive days to patients with advanced solid tumors, with attempt at dose intensification using recombinant granulocyte colony-stimulating factor. *J Natl Cancer Inst* **85**: 1499–1507

Verweij J, Lund B, Beijnen J, Planting A, De Boer DM, Koier J, Rosing H and Hansen H (1993) Phase I and pharmacokinetics study of topotecan, a new topoisomerase I inhibitor. *Ann Oncol* **4**: 673–678

Weitz JJ, Jung SH, Marschke RJ, Fitch TR and Jett JR (1995) Randomized Phase II trial of two schedules of Topotecan for the treatment of advanced stage non-small cell lung carcinoma (NSCLC): A North Central Cancer Treatment Group (NCCTG) Trial. *Proc Annu Meet Am Soc Clin Oncol* **14**: A1053

© Cancer Research Campaign 1997